# Health insurance’s contribution to reducing the financial burden of tuberculosis in Guizhou Province, China

**DOI:** 10.1017/S0950268824001316

**Published:** 2024-12-11

**Authors:** Rong Du, Xiaoxue Ma, Aiju Huang, Huijuan Chen, Xueli Guo, Jian Zhou, Jinlan Li, Weibing Wang, Qi Zhao

**Affiliations:** 1 School of Public Health, Fudan University, Shanghai, China; 2 NHC Key Laboratory of Health Technology Assessment, Fudan University, Shanghai, China; 3 Guizhou Center for Disease Control and Prevention, Guizhou, China

**Keywords:** catastrophic health expenditure, equality, social health insurance, socioeconomic strata, tuberculosis

## Abstract

Despite global efforts to end tuberculosis (TB), the goal of preventing catastrophic health expenditure (CHE) due to TB remains unmet. This cross-sectional study was conducted in Guizhou Province, Southwest China. Data were collected from the Hospital Information System and a survey of TB patients who had completed standardized antituberculosis treatment between January and March 2021. Among the 2 283 participants, the average total expenditure and out-of-pocket expenditure were $1 506.6 (median = $760.5) and $683.6 (median = $437.8), respectively. Health insurance reimbursement reduced CHE by 16.8%, with a contribution rate of 24.9%, and the concentration index changed from -0.070 prereimbursement to -0.099 postreimbursement. However, the contribution of health insurance varied significantly across different economic strata, with contribution rates of 6.4% for the lowest economic group and 53.1% for the highest group. For patients from lower socioeconomic strata, health insurance contributed 10.7% to CHE in the prediagnostic phase and 23.5% during treatment. While social health insurance alleviated the financial burden for TB patients, it did not provide sufficient protection for those in lower economic strata or during the prediagnostic stage. This study underscores the need for more effective and equitable subsidy policies for TB patients .

## Introduction

Tuberculosis (TB) poses a significant threat to public health and imposes substantial socioeconomic challenges [1]. Universal health coverage (UHC) aims to ensure that everyone receives quality health services without experiencing financial hardship [2]. To achieve UHC, the implementation of social health insurance has been advocated to protect enrollees from financial risks and enhance the utilization of healthcare services [3–5]. Despite global efforts to eliminate TB, the goal of preventing catastrophic health expenditure (CHE) for individuals and families affected by TB remains unmet [6]. Previous studies indicate that health programmes often benefit the wealthy more than the poor [7]. In China, the current health insurance policy provides the same reimbursement rate for patients across different socioeconomic strata, which may not adequately address the financial disparities faced by poorer patients [8].

This cross-sectional survey sought to analyze the financial burden on TB patients and its determinants, with a particular focus on quantifying the impact of different health insurance schemes on mitigating the economic burden across varying socioeconomic strata and stages of diagnosis and treatment. The findings of this study aid in formulating targeted TB subsidy policies to alleviate the economic burden on TB patients .

## Methods

### Study sites

Guizhou Province, located in Southwest China, is characterized by a high prevalence of TB and relatively modest economic development. In 2020, the TB notification rate in Guizhou was 96.54 per 100 000, the third highest in China. In comparison, the gross domestic product (GDP) per capita in Guizhou was $2 746.8, significantly lower than the national average of $11 094.0.

### Study design, participants, and sample size

The cross-sectional study was conducted across 89 counties and districts in Guizhou Province from January to March 2021. The study subjects comprised drug-sensitive TB patients registered in the National Tuberculosis Information Management System (TBIMS) who had completed a standardized course of anti-TB treatment before the investigation.

Assuming 70% of TB patients incurred CHE on TB care [9], with an allowable error of 2%, a confidence interval of 95%, and accounting for a nonresponse rate of 10%, the final sample size was determined to be 2 219. The sample size of each county and district was determined using probability proportional to size (PPS), based on the registered cases in 2020 as a proportion of the total cases in the province.

### Data collection

We analyzed the financial burden of TB patients by integrating medical records from TB-designated hospitals with data from patient surveys. Following the WHO ‘Patient Cost Survey Handbook’ recommendations [10], the study gathered information on both direct and indirect costs incurred by TB patients prior to diagnosis and throughout treatment. Direct medical costs, including registration fees, examination fees, medication costs, and hospitalization fees, were extracted from the hospital HIS. The structured questionnaires were administered by trained interviewers in face-to-face interviews after patients had completed standardized anti-TB treatment. These questionnaires captured information on direct nonmedical costs (including nutrition, accommodation, transportation expenses, and others), indirect costs (including lost workdays and daily wages of patients and their family members), and basic demographic characteristics (including gender, age, type of health insurance, annual household income, and others).

### Data analysis

Epidata 3.1 was used for double-entry and validation of collected data, while SPSS 25.0 was employed for data analysis. Descriptive statistics, including mean and median, were used to summarize direct medical expenses, direct nonmedical expenses, out-of-pocket (OOP) expenditures, and total expenses across various stages of TB diagnosis and treatment. Additionally, reimbursement amount, reimbursement proportions, and the contribution rate of medical insurance were calculated to evaluate the extent of medical insurance reimbursement for patients. The Kruskal–Wallis test was performed to compare the incidence of CHE among patients of different socioeconomic strata, with a *p*-value of <0.05 considered significant. Binary logistic regression was utilized to examine the determinants of CHE. Concentration indices (CIs) were used to assess the equity of CHE distribution among TB patients with varying levels of medical insurance coverage across different socioeconomic strata before and after reimbursement.

### Definitions

Total expenditure for TB treatment encompasses all costs from symptom onset to treatment completion, divided into direct medical, direct nonmedical, and indirect expenditures. Direct medical costs include expenses for diagnosis and treatment, while direct nonmedical costs cover transportation, accommodation, food, and other related expenses. Indirect costs represent income losses for patients and their families. OOP expenditures are the costs that patients pay themselves, covering direct medical and nonmedical expenses, but excluding indirect costs.

CHE occurs when households must sacrifice basic needs, sell assets, incur debt, or otherwise become poorer due to OOP expenditures surpassing a specific percentage of their income or total expenditure [3, 11]. In this study, CHE was defined as TB-related OOP expenses exceeding 10% of a household’s annual income.

The reimbursement proportion is defined as the percentage of medical expenses reimbursed by insurance relative to the direct medical expenditures incurred before reimbursement.

The contribution rate quantifies the extent to which medical insurance alleviates the financial burden of care. It is calculated as the difference between prereimbursement and postreimbursement CHE rates, divided by the initial prereimbursement CHE rate.

Autonomous prefecture: A prefecture-level administrative region in China, governed by Guizhou Province, established for ethnic minorities with a high degree of self-governance.

Extra subsidies: Additional financial assistance from the government for impoverished families, including those unable to work, without a means of livelihood, disabled individuals, minors, and those below the national poverty line.

Hospitalization refers to inpatient treatment during the antituberculosis therapy period.

Delay in diagnosis: The interval between a patient’s first medical visit for TB symptoms and the confirmed diagnosis exceeding 14 days [12].

Diagnosis and treatment time is divided into two phases: the prediagnosis stage, which spans from the onset of suspected TB symptoms to diagnosis confirmation, and the treatment stage, which encompasses the duration of standardized TB treatment.

The insurance schemes examined in this study encompass four types [13, 14], as shown in Table 1.

**Table 1.** Definitions and reimbursement rates of four medical insurance schemes

	UEBMI	URRMI	URBMI	NRCMS
Eligible population	Urban, employed	Urban, rural, nonemployed	Urban, nonemployed	Rural, employed, and nonemployed
Source of funding	Contributory (8% of annual payroll, 6% from employers, and 2% from employees)	integrate URBMI and NRCMS as the URRMI scheme, to achieve universal health coverage	Government subsidy (70%) and individual premium (30%)	Government subsidy (80%) and individual premium (20%)
Outpatient	79%	78%	58%	72%
Inpatient	85%	78%	78%	77%

*Note*: Outpatient and inpatient represent the outpatient and inpatient reimbursement rates of health insurance for TB patients, respectively. Reimbursement rates for different medical insurance schemes are calculated based on the average reimbursement rates across various counties and districts. NRCMS, New Rural Cooperative Medical Scheme; UEBMI, Urban Employee Basic Medical Insurance; URBMI, Urban Resident Basic Medical Insurance; URRMI, Urban-Rural Resident Medical Insurance.

### Ethics approval and consent to participate

Ethical approval for this study was granted by the Ethical Review Board of the Guizhou Centers for Disease Control and Prevention. Written informed consent was obtained from all patients prior to data collection, and the consent forms are available upon request.

## Results

### Demographic characteristics of TB patients

A total of 2 521 questionnaires were distributed. After excluding 238 invalid responses, 2 283 valid questionnaires were included in the final analysis, resulting in an effective response rate of 90.56%. The average age was 43.9 years (SD = 20.3). A total of 61.2% were male, and 69.5% were married. Of whom, 24.9% (*n* = 569) had completed a high school education or above, while 16.2% (*n* = 370) had no school education. The average annual household income was $5 751.4, varying from $2 311.2 to $7 704.2. Nearly all patients (98.0%) had national basic medical insurance, with the majority (1 617, 70.8%) enrolled in URRMI (Table 2).

**Table 2.** Binary logistic regression analysis of catastrophic health expenditure (CHE) among TB patients at different stages of diagnosis and treatment (*N* = 2 283)

		*N*	%	Prediagnosis		During treatment		The entire process	
Variables				OR (95% CI)	*P*-value	OR (95% CI)	*P*-value	OR (95% CI)	*P*-value
Gender	Female	886	38.8	–		1		–	
	Male	1 397	61.2	–		0.85 (0.66–1.09)	0.207	–	
Age groups (years)	<30	780	34.2	1		1		1	
	<45	338	14.8	0.99 (0.62–1.58)	0.972	1.06 (0.64–1.77)	0.815	1.30 (0.85–1.98)	0.230
	<60	552	24.2	1.21 (0.76–1.92)	0.413	0.93 (0.55–1.58)	0.796	1.48 (0.96–2.28)	0.073
	≥60	613	26.9	1.57 (0.97–2.54)	0.068	1.11 (0.64–1.94)	0.706	1.70 (1.08–2.66)	0.021
Marital status	Unmarried	697	30.5	1		1		1	
	married	1 586	69.5	1.02 (0.68–1.53)	0.927	1.06 (0.68–1.65)	0.809	1.06 (0.72–1.55)	0.770
Education	Not receive schooling	370	16.2	1		1		1	
	Primary school	682	29.9	1.43 (1.05–1.93)	0.021	1.23 (0.85–1.76)	0.270	1.43 (1.04–1.97)	0.026
	Junior high school	662	29.0	1.14 (0.80–1.62)	0.467	1.41 (0.92–2.14)	0.111	1.65 (1.16–2.35)	0.006
	High school and above	569	24.9	0.89 (0.57–1.40)	0.620	1.35 (0.82–2.24)	0.239	1.43 (0.94–2.15)	0.091
Ethnicity	Han nationality	1 270	55.6	1		1		1	
	Minority	1 013	44.4	0.96 (0.76–1.19)	0.691	0.82 (0.64–1.06)	0.127	0.84 (0.68–1.04)	0.102
Autonomous prefecture	Yes	1 055	46.2	1		1		1	
	No	1 228	53.8	1.27 (1.01–1.58)	0.038	1.49 (1.16–1.92)	0.002	1.40 (1.14–1.74)	0.002
Annual household income, quartile	Q4 (highest)	483	21.2	1		1		1	
	Q3 (high)	524	23.0	2.69 (1.79–4.05)	0.001	3.64 (2.21–5.98)	0.001	3.55 (2.59–4.86)	0.001
	Q2 (low)	701	30.7	4.51 (3.08–6.59)	0.001	5.97 (3.74–9.55)	0.001	7.34 (5.43–9.92)	0.001
	Q1 (lowest)	575	25.2	11.05 (7.51–16.26)	0.001	31.10 (19.29–50.12)	0.001	37.35 (25.96–53.72)	0.001
Insurance	UEBMI	119	5.2	1		1		–	
	URRMI	1 617	70.8	1.33 (0.74–2.39)	0.348	0.46 (0.26–0.81)	0.007	–	
	URBMI	64	2.8	1.05 (0.51–2.16)	0.897	0.58 (0.29–1.17)	0.126	–	
	NRCMS	474	20.8	0.97 (0.56–1.70)	0.929	0.33 (0.19–0.56)	<0.001	–	
	Other insurance	9	0.4	2.08 (0.88–4.94)	0.096	0.33 (0.12–0.87)	0.026	–	
Extra subsidies	No	1 787	78.3	1		1		1	
	Yes	496	21.7	0.89 (0.69–1.14)	0.347	1.03 (0.78–1.36)	0.824	0.85 (0.66–1.09)	0.191
Hospitalization	No	1 635	71.6	–		1		1	
	Yes	648	28.4	–		3.78 (2.96–4.83)	<0.001	1.85 (1.48–2.30)	<0.001
Delay in diagnosis	No	1 582	69.3	1		–		1	
	Yes	701	30.7	1.71 (1.34–2.19)	<0.001	–		1.54 (1.20–1.98)	<0.001

*Note*: ‘–’ indicates that the variable was not included in the regression analysis. NRCMS, New Rural Cooperative Medical Scheme; UEBMI, Urban Employee Basic Medical Insurance; URBMI, Urban Resident Basic Medical Insurance; URRMI, Urban-Rural Resident Medical Insurance.

### The financial burden of TB patients and its determinants

After insurance reimbursement, the average total expenditure for TB patients was $1 506.6 (median = $760.5) and OOP expenses were $683.6 (median = $437.8) (Table 3). Indirect costs and direct medical costs accounted for 54.6% and 34.8% of the average total expenditure, respectively, higher than the 10.6% for direct nonmedical costs. Prediagnosis and during-treatment OOP expenses accounted for 53.8% and 46.2% of the total OOP expenses, respectively. Compared to patients with higher socioeconomic status, those with lower socioeconomic status had significantly lower total expenditures ($1 236.5 vs. $2 002.3, *p* < 0.001) and OOP expenses ($596.5 vs. $761.9, *p* < 0.001). Binary logistic regression indicated that age (OR = 1.70, 95% CI = 1.08–2.66, *P* = 0.021), education (OR = 1.65, 95% CI = 1.16–2.35, *P* = 0.006), residence in autonomous regions (OR = 1.40, 95% CI = 1.14–1.74, *P* = 0.002), hospitalization (OR = 1.85, 95% CI = 1.48–2.30, *P* < 0.001), and diagnostic delay (OR = 1.54, 95% CI = 1.20–1.98, *P* < 0.001) were significantly associated with CHE. Patients with the lowest economic status had a significantly higher risk of CHE prediagnosis (OR = 11.05, 95% CI = 7.51–16.26, *P* = 0.001), during treatment (OR = 31.10, 95% CI = 19.29–50.12, *P* = 0.001), and overall (OR = 37.35, 95% CI = 25.96–53.72, *P* = 0.001) compared to those with higher economic status (Table 2).

**Table 3.** Financial burden among TB patients of different socioeconomic strata at various diagnosis and treatment stages

	Total (*n* = 2 283)		Q1 (*n* = 575)		Q2 (*n* = 701)		Q3 (*n* = 524)		Q4 (*n* = 483)		
	Mean	Median	Mean	Median	Mean	Median	Mean	Median	Mean	Median	*P*-value
Prediagnosis											
Direct nonmedical expenditure	97.2	26.2	93.7	26.2	87.2	18.5	104.8	35.7	107.8	30.8	0.108
Direct medical expenditure	270.7	97.3	217.6	77.6	249.7	77.3	320.6	120.0	310.2	132.5	<0.001
Out-of-pocket (OOP)	367.9	155.6	311.3	127.3	336.9	117.1	425.3	173.0	417.9	198.2	0.002
During treatment											
Direct nonmedical expenditure	61.4	12.3	74.6	13.9	57.2	12.3	63.3	12.3	49.7	10.8	0.011
Direct medical expenditure	254.3	106.3	228.2	93.2	236.7	92.6	264.2	115.9	300.2	129.8	<0.001
OOP	315.7	137.4	302.8	124.0	294.0	127.4	327.4	141.6	349.9	162.5	0.010
The entire process											
Direct nonmedical expenditure (*a*)	158.6	88.4	150.7	87.8	163.4	95.5	167.5	84.7	151.5	83.8	0.855
Direct medical expenditure (*b*)	525.0	278.5	445.8	237.4	486.5	257.8	584.8	312.0	610.4	320.3	<0.001
OOP (*a*) + (*b*)	683.6	437.8	596.5	396.3	649.8	416.0	752.2	468.2	761.9	492.3	<0.001
Indirect expenditure (*c*)	823.0	0	640.0	0	665.8	0	849.4	14.3	1 240.4	77.0	0.001
Total expenditure (*a*) + (*b*) + (*c*)	1 506.6	760.5	1 236.5	651.6	1 315.7	721.1	1 601.6	903.3	2 002.3	919.5	<0.001

*Note*: The average exchange rate of the Chinese Yuan to the U.S. Dollar from January to March 2021 was approximately 6.49 CNY per 1 USD ($1 = 6.49 CNY). Q1 to Q4 represent different socioeconomic strata based on quartiles of patients’ self-reported annual household income: Q1, lowest; Q2, low; Q3, high; Q4, highest.

### Health insurance’s contribution to reducing the financial burden

The average reimbursement amount from health insurance was $584.0 (median: $291.8), covering 45.8% of direct medical costs. The reimbursement proportion was higher during treatment than in the prediagnosis phase (38.5% vs. 33.8%), despite the higher average reimbursement amount before diagnosis ($343.3 vs. $240.8). There were no significant differences in the amount (*P* = 0.088) and proportion (*P* = 0.911) of reimbursements among patients of different socioeconomic levels (Table 4). CHE decreased from 67.4% before reimbursement to 50.6% after reimbursement, with a reduction of 13.2% before diagnosis and 9.9% during treatment. Overall, patients with lower socioeconomic status experienced higher CHE both before and after insurance reimbursement compared to those with higher socioeconomic status. Across all stages – prediagnosis, during treatment, and overall – health insurance provided a higher contribution rate to patients with higher socioeconomic status (Table 4).

**Table 4.** Medical insurance reimbursement and catastrophic health expenditure (CHE) for TB patients with different socioeconomic strata at various stages of diagnosis and treatment

	Medical insurance reimbursement amounts (USD)		Medical insurance reimbursement proportions (%)		CHE		Contribution rate (*c*)
	Mean	Median	Mean	Median	Before (*a*)	After (*b*)	
Prediagnosis							
Total (*n* = 2 283)	343.3	33.9	33.8	33.1	40.4	27.2	32.7
Q1 (*n* = 575)	311.5	18.8	33.2	20.0	56.3	50.3	10.7
Q2 (*n* = 701)	313.7	30.8	33.2	30.0	42.4	28.1	33.7
Q3 (*n* = 524)	388.4	53.6	35.3	37.5	37.6	18.3	51.3
Q4 (*n* = 483)	375.1	46.2	33.5	32.5	21.7	8.3	61.8
*P*-value	0.235		0.670		<0.001	<0.001	–
During treatment							
Total	240.8	55.2	38.5	37.6	33.1	23.2	29.9
Q1	198.2	35.5	37.8	34.9	67.3	51.5	23.5
Q2	227.0	45.5	37.6	35.7	29.4	19.5	33.7
Q3	267.8	61.7	40.3	42.0	19.8	13.0	34.3
Q4	282.1	74.0	38.5	39.3	12.2	5.8	52.5
*P*-value	0.002		0.330		<0.001	<0.001	–
The entire process							
Total	584.0	291.8	45.8	50.2	67.4	50.6	24.9
Q1	509.7	266.1	46.2	52.3	92.0	86.1	6.4
Q2	540.7	231.2	45.6	50.6	73.2	55.1	24.7
Q3	656.2	322.8	45.9	50.0	62.4	37.2	40.4
Q4	657.2	311.7	45.5	50.0	35.0	16.4	53.1
*P*-value	0.088		0.911		<0.001	<0.001	–

*Note*: The average exchange rate of the Chinese Yuan to U.S. Dollar from January to March 2021 was approximately 6.49 CNY per 1 USD ($1 = 6.49 CNY). Q1 to Q4 represent different socioeconomic strata based on quartiles of patients’ self-reported annual household income: Q1, lowest; Q2, low; Q3, high; Q4, highest. ‘Before’ and ‘After’, respectively, represent the incidence rate of CHE before and after medical insurance reimbursement; Contribution rate (*c*) = ((*a*) - (*b*))/(*a*).

### Equity in the distribution of CHE due to social health insurance

**Figure 1.**
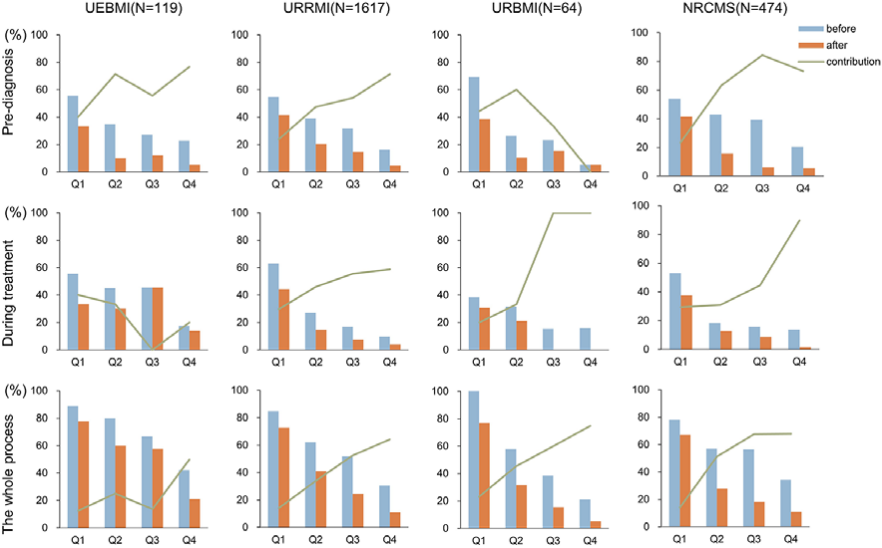
Comparison of catastrophic health expenditure (CHE) before and after medical insurance reimbursement across different socioeconomic strata and insurance types. Q1 to Q4 represent different socioeconomic strata based on quartiles of patients’ self-reported annual household income: Q1, lowest; Q2, low; Q3, high; Q4, highest. “Before” represents the incidence rate of CHE before medical insurance reimbursement (a); “After” represents the incidence rate of CHE after medical insurance reimbursement (b); the Contribution rate is calculated as ((a) - (b)) / (a). UEBMI, Urban Employee Basic Medical Insurance; URRMI, Urban-Rural Resident Medical Insurance; URBMI, Urban Resident Basic Medical Insurance; NRCMS, New Rural Cooperative Medical Scheme.

When stratified by insurance reimbursement status, type of insurance, and diagnosis and treatment stage, the CI of CHE was consistently negative and statistically significant (*P* < 0.05) (Table 5). This finding indicates that, both before and after insurance reimbursement, CHE was concentrated among households with lower socioeconomic status, showing a pro-poor pattern. After reimbursement, the absolute value of CI decreased during the treatment stage, suggesting a more equitable distribution of CHE among the population. However, the absolute value of CI increased after reimbursement during the prediagnosis stage and the entire process, indicating a higher concentration of CHE among lower socioeconomic households. This trend was consistent across different types of health insurance and throughout the entire period. In terms of the contribution rate of health insurance to reducing CHE, all types of insurance showed a higher contribution to patients with higher socioeconomic status before diagnosis, during treatment, and overall (Figure 1).

**Table 5.** Concentration indices (CIs) for catastrophic health expenditure (CHE) before and after medical insurance reimbursement due to TB diagnosis and treatment (*n* = 2 283)

	CHE before reimbursement		CHE after reimbursement	
	CI	*P*-value	CI	*P*-value
Total (*n* = 2 283)				
Prediagnosis	–0.051	<0.001	–0.070	<0.001
During treatment	–0.088	<0.001	–0.079	<0.001
The entire process	–0.070	<0.001	–0.099	<0.001
UEBMI (*n* = 119)				
Prediagnosis	–0.039	0.036	–0.047	0.006
During treatment	–0.068	<0.001	–0.051	0.006
The entire process	–0.083	<0.001	–0.104	<0.001
URRMI (*n* = 1 617)				
Prediagnosis	–0.054	<0.001	–0.071	<0.001
During treatment	–0.097	<0.001	–0.088	<0.001
The entire process	–0.072	<0.001	–0.102	<0.001
URBMI (*n* = 64)				
Prediagnosis	–0.086	0.001	–0.074	0.002
During treatment	–0.043	0.091	–0.069	0.002
The entire process	–0.100	<0.001	–0.113	<0.001
NRCMS (*n* = 474)				
Prediagnosis	–0.033	<0.001	–0.065	<0.001
During treatment	–0.074	<0.001	–0.067	<0.001
The entire process	–0.055	<0.001	–0.092	<0.001

*Note*: NRCMS, New Rural Cooperative Medical Scheme; UEBMI, Urban Employee Basic Medical Insurance; URBMI, Urban Resident Basic Medical Insurance; URRMI, Urban-Rural Resident Medical Insurance.

## Discussion

Our research revealed that TB patients in the surveyed area still face a heavy economic burden, with an average total expenditure of $1 506.6 after health insurance reimbursement, higher than the national average of $1 022.0 [15]. Although medical insurance has somewhat reduced the risk of CHE for TB patients, nearly half of the surveyed subjects still incurred catastrophic expenditures. The guarantee levels of different types of medical insurance are different, and the degrees of reduction also vary. Improving the equity of health insurance benefits is essential to ensure adequate financial protection for all patients.

Despite having lower OOP expenditures and total expenditures, patients with lower socioeconomic status are more prone to CHE [16, 17]. Patients with higher socioeconomic status are more likely to leverage their greater financial resources to access healthcare services not covered by insurance, such as additional diagnostic tests, more expensive medications, and other supplementary treatments [18]. Conversely, patients with lower socioeconomic status tend to reduce the frequency of medical visits to minimize expenses, resulting in relatively lower total and OOP costs [19]. However, they remain more vulnerable and sensitive to economic burdens [20, 21]. With no significant difference in reimbursement amounts and proportions among different socioeconomic groups, patients with better socioeconomic status require less subsidy to avoid CHE. Meanwhile, post-reimbursement OOP expenses still exceed the financial capacity of lower socioeconomic status patients, leading to CHE concentration in these households [13, 20]. In terms of insurance type, higher-income groups predominantly use UEBMI, while lower-income groups mainly use URBMI and NRCMS. UEBMI offers a relatively higher reimbursement proportion for outpatient and inpatient care [22, 23]. Moreover, current health insurance primarily reimburses a portion of direct medical costs with limited coverage and reimbursement proportions. Unnecessary medications and additional tests contribute to high OOP expenses [24]. Additionally, indirect costs such as lost wages and unemployment, which are significant contributors to high total costs, are not accounted for in the current insurance scheme [25]. Although some regions in Guizhou provide additional support for elderly and disabled families, these subsidies are insufficient to prevent CHE.

During the diagnostic stage, patients incur higher OOP expenses and rates of CHE compared to the treatment stage. When patients lack adequate awareness of TB, they often seek care at non-designated TB facilities. These facilities may fail to promptly identify typical TB symptoms and provide standard diagnostic tests, leading to additional, unnecessary examinations [20]. Simultaneously, it increases the frequency of medical visits, resulting in higher transportation, food, and accommodation costs [19]. These factors can elevate the risk of delayed diagnosis, which is a significant contributor to CHE in the prediagnosis stage. Additionally, during this period, both patients and their caregivers face prolonged absences from work or even unemployment, further intensifying the economic burden [14, 26–29]. Hospitalization during treatment is a key factor in the occurrence of CHE, consistent with multiple studies [16, 30]. Even though some insurance schemes, such as NRCMS, offer higher reimbursement rates during hospitalization, the high cost of repeated hospitalizations increases overall OOP expenses [22].

In the context of UHC, China’s health insurance has only limitedly reduced the economic burden for TB patients and fails to provide sufficient protection to prevent CHE, especially for those with lower socioeconomic status [26]. Therefore, prioritizing TB and other key health interventions is necessary to provide quality healthcare services, alleviate the economic burden, and prevent CHE among TB patients [25]. First, improve standardized diagnosis and treatment processes for TB to reduce unnecessary expenditures from delayed diagnosis; second, strengthen regulatory processes to avoid unnecessary medications, tests, and hospitalizations; third, develop economic status evaluation metrics for TB patients to provide additional subsidies to those with lower socioeconomic status; and fourth, optimize TB financing policies to increase reimbursement amounts and proportion for TB patients.

## Limitations

This study has several limitations. First, indirect costs and direct nonmedical expenses were self-reported by patients, which could introduce recall bias. Additionally, the study excluded patients who did not receive standardized treatment or discontinued treatment, potentially leading to an underestimation of CHE, as these individuals may have abandoned treatment due to limited financial capacity.

## Conclusion

This study reveals that CHE is predominantly concentrated among TB patients with lower socioeconomic status. The contributions of various types of health insurance to TB patients vary significantly across different socioeconomic levels, with higher contributions observed for patients with higher socioeconomic status. This disparity is evident before diagnosis, during treatment, and throughout the entire process. In the context of achieving UHC, prioritizing key health interventions for TB, optimizing TB financing policies, and focusing on patients with lower socioeconomic status are crucial. These measures are essential to effectively reduce CHE and promote health equity.

## Data Availability

The data that support the findings of this study are available from the Guizhou Center for Disease Control and Prevention. Restrictions apply to the availability of these data, which were used under licence for this study. Data are available from the corresponding author with the permission of the Guizhou Center for Disease Control and Prevention.

## Abbreviations

CDC: Center for Disease Control and Prevention
CHE: Catastrophic Health Expenditure
CI: Concentration Index
GDP: Gross Domestic Product
HIS: Hospital Information System
NRCMS: New Rural Cooperative Medical Scheme
OOP: Out-of-pocket
TB: Tuberculosis
TBIMS: National Tuberculosis Information Management System
UEBMI: Urban Employee-based Basic Medical Insurance Scheme
UHC: Universal Health Coverage
URBMI: Urban Resident-based Basic Medical Insurance
URRMI: Urban-Rural Resident Medical Insurance
